# Understanding Customers’ Continuance Intentions Toward In-Lobby Self-Service Technologies

**DOI:** 10.3389/fpsyg.2019.00332

**Published:** 2019-02-19

**Authors:** Mingfei Li, Shanshan Huang

**Affiliations:** ^1^Business School, Sichuan University, Chengdu, China; ^2^School of Economics and Management, Hubei University for Nationalities, Enshi, China

**Keywords:** self-service technology, in-lobby, customer continuance intention, customer perceived service climate, customer satisfaction, customer readiness

## Abstract

Drawing on service climate theory and insights from the literature on self-service technologies (SSTs) and customer participation, this study investigates the antecedents of customers’ continuance intentions toward in-lobby SSTs. Using data collected from 257 actual customers in the context of retail banks, this experimental study examines the proposed relationship between customer perceived service climate, customer readiness factors (i.e., perceived ability, role clarity, and perceived benefit), customer satisfaction and customer continuance intention toward in-lobby SSTs. The results show that customers’ perceived service climate positively influences customers’ continuance intentions toward in-lobby SSTs. Moreover, two customer readiness factors (i.e., perceived ability, perceived benefit) and customer satisfaction mediate this relationship. The findings demonstrate the importance of customers’ perceived service climate in driving their continuance intention and provide managerial implications for service firms employing in-lobby SSTs.

## Introduction

Over the past two decades, the infusion of self-service technologies (SSTs) has changed the nature of service and how value is created (Bitner et al., 2000; Vargo and Lusch, 2004). Services are increasingly being provided to customers through the use of SSTs. The retail bank industry is at the front of this trend. Since 2011, JP Morgan Chase bank has begun to deploy new self-service kiosks (SSKs) to its outlets. Approximately 90% of transactions performed by tellers can now be handled by these SSKs. The biggest bank in China, the Industrial and Commercial Bank of China (ICBC, 2017), has equipped 60% of its branches with intelligent teller machines, through which more than 90% of teller services can be provided. The proliferation of SSTs in service industries is based on the premise that successful SSTs benefit both service providers and customers. Specifically, SSTs increase service system productivity and reduce operational costs (Bitner et al., 2000; Curran et al., 2003), while enhancing customer experience and decreasing waiting time (Wang, 2012). However, researchers have noted that not all customers embrace SSTs (Meuter et al., 2003; Reinders et al., 2008). Research focusing on drivers of the use SSTs has found that there are two categories of antecedents to customers’ use of SSTs, namely, SST characteristics and individual difference factors (Meuter et al., 2005; Wang et al., 2013). SSTs’ characteristic factors include ease of use (Lin et al., 2007; Weijters et al., 2007) and usefulness (Oh et al., 2013; Kim and Qu, 2014). Individual difference factors include attitude (Lin and Chang, 2011; Wang et al., 2012), perceived control (Lee and Allaway, 2002; Ding et al., 2007), perceived risk (Walker and Johnson, 2006; Kim and Qu, 2014) and fun (Dabholkar and Bagozzi, 2002).

Although extant research contributes to a better understanding of customers’ trial intention or initial use, only limited attempts have been made to investigate factors that influence customers’ continued use of SSTs (see Table 1 for a review of SST continuance studies). Compared to initial adoptions, customers’ continued use of SSTs is more critical to service firms (Schuster et al., 2015). Repeated and continued use is not only a necessary stage to customer commitment and loyalty (Bitner et al., 2002) but also a return of the substantial investment in SSTs (Ashworth, 2010). Furthermore, although substantial numbers of in-lobby SSTs have been employed by service firms (e.g., banks, hotels, fast-food restaurants, and supermarkets), extant research provides very little knowledge about the factors that drive customers’ continuance intentions toward them. This study aims to fill this gap.

**Table 1:** A chronological review of SST continuance studies.

Study	Context	Technology	Methodology	Key findings
Meuter et al., 2003	Context-free	Technology-free	Cross-sectional survey	Technology anxiety negatively influences customers’ intention to use the same SST option again
Eriksson and Nilsson, 2007	Bank	Internet banking	Cross-sectional survey	Perceived usefulness positively influences SSTs’ continuance, whereas multichannel satisfaction negatively influences SSTs continuance.
Ho and Ko, 2008	Bank	Internet banking	Cross-sectional survey	Ease of use, usefulness, cost saved, self-control impact on customer value and customer readiness, which in turn impact intention of continued use
Zhao et al., 2008	Library	Self-checkout machine	Cross-sectional experiment	Post-training self-efficacy positively influences ease of use and satisfaction. Ease of use and satisfaction increase customer intention to reuse SSTs
Chen et al., 2009	Context-free	Technology-free	Cross-sectional survey	Different factors (perceived usefulness, perceived ease of use, subjective norm, perceived behavioral control, optimism, and innovativeness) influence satisfaction, which in turn influences continuance intention toward SSTs
Wang, 2012	Convenience store	Multimedia kiosk	Cross-sectional survey	Perceived usefulness, perceived enjoyment, perceived control, perceived convenience, and customer satisfaction increase continued behavioral intention
Wang et al., 2013	Supermarket	Self-checkout kiosk	Longitudinal survey	Continued use of SSTs is initially driven by self-efficacy, then by satisfaction, and finally by habit
Schuster et al., 2015	Mental health	Internet and mobile phone	In-depth interview	Customers’ attitude, perceived behavioral control, goals, positive anticipated emotions, past behavior, and social support are key determinants of SST reusage

**Figure 1:**
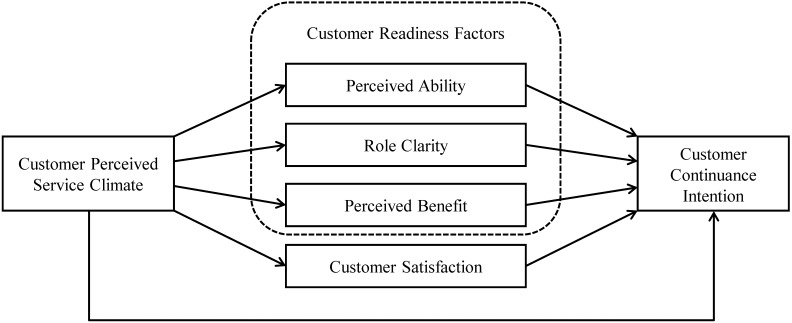
Conceptual framework of customer continuance intention toward in-lobby SSTs.

Given the unique location, customers’ experience of using in-lobby SSTs is distinct from their experience with other on-site SSTs (e.g., ATMs outside the bank lobby). Service organizations are open systems in which the managerial practices are not only for employees but also for customers (Schneider and Bowen, 1985). In-lobby customers have more opportunities to perceive service firms’ managerial practices and frontliners’ service behaviors toward SST usage. Therefore, the question that arises is as follows: Do customers’ service climate perceptions affect their SST continuance intention? Moreover, previous SST continuance research (as shown in Table 1) has found that customer satisfaction (Wang, 2012; Wang et al., 2013) and customer readiness factors (Ho and Ko, 2008; Zhao et al., 2008) mediate the relationship between antecedents (e.g., ease of use, usefulness, self-efficacy) and the continued use of SSTs. Another question that arises is the following: Do customer satisfaction and customer readiness factors mediate the relationship between customers’ service climate perceptions and continued use of SSTs? With the specified research questions in mind, this study proposes and tests a conceptual model involving customers’ perceived service climate and customer continuance intention (see Figure 1). This study proposes that customer readiness factors and customer satisfaction mediate the positive influence of customers’ perceived service climate on continuance intention toward in-lobby SSTs.

The contribution of this study is threefold. First, this study extends the SST literature by investigating the role of customers’ perceived service climate in driving customers’ continuance intentions toward in-lobby SSTs. To the best of our knowledge, this study is the first in the SST literature to link service providers’ managerial practices from the customers’ perspective to customers’ continuance intentions. Second, this study establishes the robust predictive role of perceived ability, perceived benefit and customer satisfaction in SST continuance. Third, this study empirically tests and lends support to the suggestions that Bitner et al. (2002) proposed for implementing successful SSTs.

In the following sections, this study reviews related literature to develop the conceptual framework and related hypotheses. This is followed by the methodology. This article continues by presenting the analysis and results. Finally, the study concludes by discussing implications, limitations and future research directions.

## Conceptual Framework and Hypotheses Development

### Climate Research

Climate research has a long history. Since Lewin et al. (1939) proposed the “social climate” construct in social psychology, climate research has received significant academic attention (James and Jones, 1974; Ashforth, 1985; Schneider et al., 1998, 2017; Carr et al., 2003; Parker et al., 2003).

Generally, climate refers to an individual’s perceptions of the environment. Rooted in Gestalt psychology, climate is a result of an individual’s tendency to perceive and interpret the outside world’s “order.” Individuals create their own “order” and use it to gauge the appropriateness of their behaviors (Schneider, 1975), which in turn influences their future behaviors. Consequently, the effects of climate on individual attitudes and behaviors have been investigated in many contexts (Jones and James, 1979; Ashforth, 1985), and the majority of climate studies have been conducted in the organizational behavior domain. Climate is linked to several important individual-level and organizational-level outcomes. For example, the fit between employees’ climate perceptions and personal orientations significantly affects their job satisfaction, organizational commitment and work performance (Ostroff, 1993). Employees’ service climate perceptions have a cross-level effect on customer satisfaction (Schneider et al., 1998). Using a meta-analysis approach, Parker et al. (2003) found that employees’ climate perceptions positively influence their motivation and performance. Salespeople’s organizational sale-related climate perceptions positively affect their outcome performance (Evans et al., 2007). The findings of previous research indicate that climate perceptions are significant influencers of employee performance and organizational outcomes.

### Customer Perceived Service Climate

In the seminal work of service climate research, Schneider (1973) defined climate as “the summary perception that bank customers have of their bank” based on specific service-related events and activities. Subsequently, service climate was broadened and redefined as an organization-level construct, which refers to employees’ collective sense of service quality—focused policies, practices and procedures they experience and the service quality emphasis they observe in behaviors that are rewarded, supported, and expected (Schneider et al., 1998). Because service organizations are open systems, Bowen and Schneider (2014) have indicated that service climate theory and research emphasizes the boundary-spanning effects of service climate on important customer outcomes. For example, previous studies have found that service climate significantly influences service quality (Schneider et al., 1998), customer satisfaction (Mayer et al., 2009), and customer loyalty (Liao and Chuang, 2004).

In contrast to service climate, customer perceived service climate (CPSC) refers to “a customer’s perception of the extent to which a service organization teaches, prioritizes, and recognizes outstanding customer service through organizational practices and procedures” (Jin et al., 2017). In the context of fitness services, Jin et al. (2017) found that CPSC augments the positive effect of positive customer-to-customer interaction on support from other customers while mitigating the negative effect of dysfunctional customer behavior on support from other customers.

Service providers have become the facilitators of value creation (Grönroos, 2008; Payne et al., 2008), which should create and maintain an environment to facilitate the co-creation activities of both service providers and customers. When the level of CPSC is high, customers perceive and receive more outstanding service practices that are taught, prioritized, and recognized by the service providers. Service firms and service employees will make a greater effort to facilitate customers’ in-lobby self-service process (e.g., proactive recommendation, personal assistance, user manual and user-friendly interfaces). Therefore, this study proposes that there may be positive linkages between CPSC and important service outcomes (e.g., customer satisfaction and customer loyalty).

### Customer Continuance Intention

Continuance intention refers to customers’ intention to continue using SSTs (Bhattacherjee, 2001). Customers have often been seen as “partial employees” who are increasingly involved in service production and delivery (Lengnick-Hall, 1996; Vargo and Lusch, 2004). The positive relationships between employees’ climate perceptions and organizational commitment have been examined intensively (for a review, see Parker et al., 2003). In the current research setting (i.e., in-lobby SSTs), both service and managerial practices are visible to customers. Customers have more clues about what managerial practices have been adopted and how service procedures have been conducted. Based on these clues, customers develop service-related perceptions, attitudes and behavioral intentions (Schneider and Bowen, 1985). Furthermore, both direct (Schneider et al., 1996) and indirect effects (Liao and Chuang, 2004; Hong et al., 2013) of service climate on customer loyalty have been tested by previous works. Therefore, this study proposes that a higher level of CPSC produces greater customer continuance intention toward in-lobby SSTs (i.e., customer loyalty to SSTs), as follows:

H1: CPSC positively affects customer continuance intention toward in-lobby SSTs.

### Customer Readiness

Customer readiness refers to the degree to which a customer is prepared to use SSTs (Meuter et al., 2005). In the customer participation literature, customer readiness factors (i.e., perceived ability, role clarity, and perceived benefit) have been well established (Meuter et al., 2005; Yim et al., 2012; Dong et al., 2015). Customer readiness variables are mostly modeled as antecedents or mediators of customer co-creation (Meuter et al., 2005; Dong et al., 2008). Meuter et al. (2005) suggested that successful SST co-production relies on customer readiness factors. When customers are prepared and ready to co-create, their enhanced self-efficacy, increased role clarity and perceived benefit positively affect their intention toward future co-creation (Bowen, 1986; Dong et al., 2008).

Perceived ability refers to customers’ perceived skills and knowledge that enable them to perform effectively in using SSTs (Meuter et al., 2005; Dong et al., 2008). Customer perceived ability denotes what a customer “can do” in self-service. When customers use SSTs, they should possess the necessary skills and confidence to complete the task (Meuter et al., 2005). Bitner et al. (2002) suggested that service providers should provide effective marketing communication to assist in overcoming customers’ perceptions of incapability to use SSTs. Zhao et al. (2008) found that training improves customer self-efficacy, which in turn increases customers’ satisfaction and behavioral intention toward self-checkout machines in the library setting. As partial employees, in-lobby customers know to what extent the service firms facilitate their self-service behaviors through service climate perceptions. If CPSC is high, which means that customers feel high expectancy and strong support from the service firm, their perceived ability improves. Furthermore, when customers’ self-efficacy is enhanced, their continuance intention toward in-lobby SSTs will be greater. Previous research has shown that individuals’ sense of self-efficacy plays a major role in the way they approach goals, tasks, and challenges (Bandura, 1977). More specifically, recent SST studies have shown that self-efficacy or perceived behavioral control motivates customers to reuse SSTs (Ho and Ko, 2008; Wang et al., 2013; Schuster et al., 2015). Thus, the current study proposes that CPSC positively affects customers’ perceived ability, which in turn increases customer continuance intention toward in-lobby SSTs. Hence, as shown in Figure 1, this study hypothesizes the following:

H2: The positive effect of CPSC on customer continuance intention toward in-lobby SSTs is mediated by customers’ perceived ability.

Role clarity refers to the customer’s knowledge and understanding of what to do when using SSTs (Meuter et al., 2005). When customers participate in an unfamiliar service production process, insufficient role clarity not only constrains their task-related performance but also affects service outcomes (Dong et al., 2015). When customers use in-lobby SSTs, their role as value creator requires them to complete certain tasks that were traditionally completed by service employees. Previous climate research has revealed that frontline employees’ climate perceptions have a significant positive effect on their role clarity (Coelho et al., 2010). Similarly, when CPSC is high, customers perceive more outstanding service practices (e.g., personal support and assistance, user-friendly interfaces), which will provide customers with a deeper understanding of the new roles and further clarify the activities that they should perform when using SSTs. In addition, previous studies have indicated that customer role clarity has a significantly positive effect on customers’ intention to reuse SSTs (Dong et al., 2008; Ho and Ko, 2008). When customers work as partial employees in service systems, their role clarity influences their performance as well as the ultimate outcomes of self-service, which further affects their continuance intention toward in-lobby SSTs. Therefore, this study hypothesizes as follows:

H3: The positive effect of CPSC on customer continuance intention toward in-lobby SSTs is mediated by role clarity.

Perceived benefit is defined as customers’ evaluation or appraisal of the rewards of using SSTs (Meuter et al., 2005). According to social exchange theory, when customers contribute more resources to service production, they may want to receive additional rewards as an exchange (e.g., reduced waiting time, enjoyment). Service climate research has indicated that service employees surrounded by a high service climate have the motivation to deliver superior service (Mayer et al., 2009). If in-lobby customers find that their self-service behaviors are facilitated by service providers’ outstanding managerial and service practices, the desired service outcomes (i.e., perceived benefit) will be anticipated and obtained. Further, previous research has tested the positive relationship between perceived benefit and customers’ continued use of SSTs (Dong et al., 2008; Ho and Ko, 2008). When customers find that the anticipated service outcomes are obtainable by using SSTs, they are motivated to reuse SSTs in the future. Based on this reasoning, this study hypothesizes the following:

H4: The positive effect of CPSC on customer continuance intention toward in-lobby SSTs is mediated by customers’ perceived benefit.

### Customer Satisfaction

In this study, customer satisfaction refers to the extent to which a customer derives positive feelings from SSTs (Wang, 2012). Previous research has demonstrated the positive effect of service climate on customer satisfaction (Dean, 2004; Mayer et al., 2009). Bitner et al. (2002) suggested that customers’ repeated use of and commitment to SSTs depends on their initial use experience. When customers perceive a high level service climate, the service provider’s quality-focus service practices not only enhance the self-service experience but also improve the self-service outcomes (i.e., customer satisfaction). Furthermore, the SST literature has indicated that customer satisfaction not only represents a significant self-service outcome but also plays a predicting role in SST continuance (Zhao et al., 2008; Chen et al., 2009; Wang, 2012; Wang et al., 2013). Thus, this study hypothesizes the following:

H5: The positive effect of CPSC on customer continuance intention toward in-lobby SSTs is mediated by customer satisfaction.

## Materials and Methods

### Research Design

This study used a scenario-based, between-subjects experimental design to examine the proposed model for three reasons. First, this type of research design has been widely employed in the service marketing literature (Smith et al., 1999; Dabholkar and Bagozzi, 2002; Dong et al., 2008). Second, compared with a cross-sectional survey design, an experimental design offers more robust explanations for causal relationships. Moreover, the scenario-based experimental design is immune to biases (e.g., memory lapse, rationalization tendencies, and consistency factors) that are common issues in retrospective self-report (Smith et al., 1999). Therefore, the scenario-based between-subjects experimental design is considered appropriate for the current study.

Because the bank industry has typically been aggressive in deploying new SSTs (Bhattacherjee, 2001), this study chose the retail bank as the research setting. To develop the scenarios, this study followed the procedure outlined by Dong et al. (2008). In the first step, three focus group discussions were conducted. A total of twenty-nine customers who had used the new SSKs in bank lobbies participated (48% female; mean age = 22.1 years). In each focus group session, the authors introduced the research topic and asked the participants two questions: What did the bank do to facilitate your in-lobby self-service? What factors positively/negatively affect your continuance intention toward in-lobby SSTs? Each session lasted between 35 and 55 min. All focus group sessions were recorded and transcribed. In the second step, the raw text was analyzed. In total, these discussions provided 21 factors that may influence customers’ continuance intentions. To ensure the inter-rater reliability of these factors, the authors evaluated each of them. This study only retained factors that both authors evaluated as service-climate-related. Factors that received less than two confirmations were eliminated from the initial factor pool. Then, all retained factors were ranked by frequency. The top five most frequently mentioned factors were proactive recommendation (18), personal assistance (18), user guide (16), user-friendly interface (15), and actual reduced time (12). The factors identified from actual self-service experiences further enhanced the quality of the scenarios. In addition, 62% participants reported that they used the SSK for online-banking services. Therefore, the online-banking registration was selected as the stimulus material.

This study manipulated CPSC at three levels: high, moderate and low. In the high CPSC condition, customers perceive more outstanding service practices that the bank recognizes, prioritizes and teaches to facilitate the self-service process. Proactive recommendation, available personal assistance, a concise user manual, and user-friendly interfaces are simultaneously provided. In the moderate CPSC condition, the service practices that the bank implements are limited to user-friendly interfaces and user manuals. In the low CPSC condition, the bank’s only effort is providing an in-lobby SSK with a complicated interface. The basic scenario and specific scenarios are shown in the Appendix.

### Participants and Procedure

The survey-based experimental design allowed this study to collect data from actual servicescapes (i.e., bank lobbies). Nine branches of the two biggest banks of China agreed to participate. This study invited customers who were waiting for services in bank lobbies to participate in this study. Participants were instructed to read a basic scenario that was held constant across all conditions and to immerse themselves in the situation. Imagining themselves as customers in a bank lobby, the participants were randomly assigned to one of three conditions and asked to read a specific scenario. Then, the participants completed the construct measurements, manipulation checks, and demographic questions. To minimize the possible demand effect, all items in the questionnaire were randomly ordered.

A total of 257 bank customers completed the study. The cell sizes for the three groups, i.e., low, moderate and high CPSC, were 81, 90, and 86, respectively. Among the subjects, approximately 51.8% were female. They ranged in age from 20 to 56, with a mean of 34.9. Education levels were high school or less (49.8%), associate degree (36.2%), bachelor’s degree (12.5%), and master’s degree or above (1.6%). Moreover, 42.4% of the subjects had similar self-service experience. Compared with the general population (National Bureau of Statistics of China, 2017), the sample was slightly younger and more educated. Previous studies have indicated that younger, male and more educated customers are more willing to use SSTs (Meuter et al., 2003). In addition, Meuter et al. (2005) found that previous similar experience is related to customer self-efficacy and behavioral intention toward SSTs. Thus, the current study controlled these variables (i.e., age, gender, education, and similar experience) to rule out potential confounding influences.

### Measures

All scales in this study were selected from previous research (see Table 2). Using Likert-type scales with anchors from strongly disagree (1) to strongly agree (7), customer continuance intention was measured using three items adapted from Bhattacherjee (2001) and Wang (2012), perceived ability was measured by three items adapted from Meuter et al. (2005), role clarity was measured by three items adapted from Meuter et al. (2005), perceived benefit was measured by four items adapted from Dong et al. (2015), and customer satisfaction was measured using three items adapted from Wang (2012). The original questionnaire was prepared in English and translated into Chinese by the authors; then, an English professor was invited to back translate the Chinese version into English (Brislin, 1980). The translators compared the two English versions and made adjustments to the Chinese scales. The forward and backward translation procedure helped to ensure that there was no mismatch or meaning loss after the translation process.

**Table 2:** Measures and results of the measurement model.

Construct/items	Std. loading	Cronbach’s α	CR	AVE
*Perceived ability*		0.896	0.901	0.753
I am fully capable of using this self-service kiosk	0.813			
I am confident in my ability to use this self-service kiosk	0.943			
Using this self-service kiosk is well within the scope of my abilities	0.841			
*Role clarity*		0.902	0.904	0.758
I feel certain about how to use this self-service kiosk properly	0.868			
I am not sure how to use this self-service kiosk properly (R)	0.879			
I know what is expected of me if I use the self-service kiosk	0.865			
*Perceived benefit*^∗^		0.885		
Using this self-service kiosk, I get what I really want				
Using this self-service kiosk, I get service in a timely manner				
Using this self-service kiosk brings me good quality service				
Using this self-service kiosk provides me with feelings of enjoyment				
*Customer satisfaction*		0.861	0.867	0.684
Overall, I am satisfied with the kiosk offered by the bank	0.865			
The kiosk offered by the bank exceeds my expectations	0.841			
The kiosk offered by the bank is close to my ideal SSTs	0.773			
*Customer continuance intention*		0.913	0.914	0.780
I intend to continue using this kiosk for service in the future	0.895			
I will continue using this kiosk for service in the future	0.867			
If I could, I would like to discontinue my use of this kiosk (R)	0.887			

χ^2^, df		102.530, 48		
GFI		0.943		
CFI		0.975		
NFI		0.954		
IFI		0.975		
RMSEA		0.067		

*CR, composite reliability; AVE, average variance extracted; GFI, goodness-of-fit index; CFI, comparative fit index; NFI, normed fit index; IFI, incremental fit index; RMSEA, root mean square error of approximation.*^∗^*Scale as a composite construct was not included in the measurement model (CFA).*

## Analysis and Results

### Manipulation Check

The effectiveness of the manipulation was assessed using a specific item stating, “The bank makes a considerable effort to facilitate your self-service process.” Participants responded on a Likert-type scale with anchors of strongly disagree (1) and strongly agree (7). Analysis of variance (ANOVA) results indicated that the participants’ ratings were significantly different across the three conditions: *F*(2,254) = 208.78, *p* < 0.000, M_low_ _CPSC_ = 3.14, M_moderate_ _CPSC_ = 4.85, and M_high_ _CPSC_ = 5.90. Therefore, the manipulation of CPSC was successful.

### Measurement Model

CPSC, as a manipulated experimental variable, was not included in the measurement model. In addition, perceived benefit, as a formative latent construct with four different aspects (Dong et al., 2015), was excluded from the CFA model. Thus, this study input perceived ability, role clarity, customer satisfaction, and continuance intention into the CFA.

As shown in Table 2, the CFA results revealed a satisfactory model fit. Six commonly used fit indexes were calculated: χ^2^(48) = 102.530, GFI = 0.943, CFI = 0.975, NFI = 0.954, IFI = 0.975 and RMSEA = 0.067. Considering the reliability, the composite reliability (CR) for each scale exceeded 0.70, and all Cronbach’s α coefficients of the scales were above 0.70 (from 0.861 to 0.913). Moreover, all standardized factor loadings were sufficiently large and significant (all *p*-values less than 0.001). Convergent validity was confirmed by the internal structure of the CFA and average variance extracted (AVE) of the scales. All items loaded on their respective constructs. All estimated AVEs of the scales ranged from 0.684 to 0.780, which were greater than the recommended level of 0.50 (Bagozzi and Yi, 1988). Discriminant validity was supported by comparisons of the correlation between two constructs to the respective square root of AVE of each construct in the pair (Fornell and Larcker, 1981). The results of the comparisons (see Table 3) indicated satisfactory discriminant validity. Therefore, this study concluded that the measurement model, which possessed sufficient reliability and validity, fit the data well.

**Table 3:** The means, SDs, and correlation coefficients.

	Mean	SD	1	2	3	4	5	6	7	8	9
(1) Gender	0.48	0.50	NA								
(2) Age	34.88	8.67	0.082	NA							
(3) Education	1.66	0.76	0.036	-0.084	NA						
(4) Experience	0.42	0.50	-0.041	-0.121	0.118	NA					
(5) Perceived ability	4.53	1.05	0.043	-0.101	0.056	0.103	**0.868**				
(6) Role clarity	4.25	1.25	-0.107	-0.073	0.066	0.114	0.341^∗∗∗^	**0.871**			
(7) Perceived benefit	4.48	1.02	-0.037	-0.008	0.058	0.174^∗∗^	0.472^∗∗∗^	0.415^∗∗∗^	NA		
(8) Customer satisfaction	5.17	0.85	0.094	-0.053	0.057	0.198^∗∗^	0.479^∗∗∗^	0.185^∗∗^	0.435^∗∗∗^	**0.827**	
(9) Customer continuance intention	5.24	0.98	0.095	0.049	0.018	0.121	0.571^∗∗∗^	0.269^∗∗∗^	0.590^∗∗∗^	0.554^∗∗∗^	**0.883**

*N = 257; ^∗^p < 0.05; ^∗∗^p < 0.01; ^∗∗∗^p < 0.001. NA, not available. The square roots of the AVE values are shown as bold at diagonal.*

### Hypotheses Testing

An ANCOVA was run on customer continuance intention, with gender, age, education and similar experience as covariates. The results revealed a significantly positive effect of CPSC on customer continuance intention, *F*(2,250) = 121.89, *p* < 0.000. Pairwise comparisons between the means of customer continuance intention indicated that participants assigned to the high CPSC group (M_high_ _CPSC_ = 6.00) had a significantly higher continuance intention than those assigned to the moderate CPSC group (M_moderate_ _CPSC_ = 5.36) or the low CPSC group (M_low_ _CPSC_ = 4.31). Furthermore, participants who were assigned to the moderate CPSC group had a higher continuance intention on average than those who were assigned to the low CPSC group. Thus, H1 was supported. With regard to the covariates, the results showed that, in contrast to age (*p* = 0.014) and gender (*p* = 0.038), education and similar experience had no significant effects on customer continuance intention. The covariates in this analysis purified the effect of CPSC on customer continuance intention.

The mediation analysis was conducted with the PROCESS macro (Hayes, 2013; Hayes and Preacher, 2014) using Model 4 with 5,000 bootstrap samples. Because the independent variable in the current study was manipulated at three levels, Hayes’ method was appropriate for detecting the proposed mediating effects. Additionally, Hayes and Preacher (2014) advocated constructing asymmetric bootstrap confidence intervals (CIs) for indirect effect (IE) statistical inference, which does not need to meet the hypothesis of the normality distribution of indirect effects.

**Figure 2:**
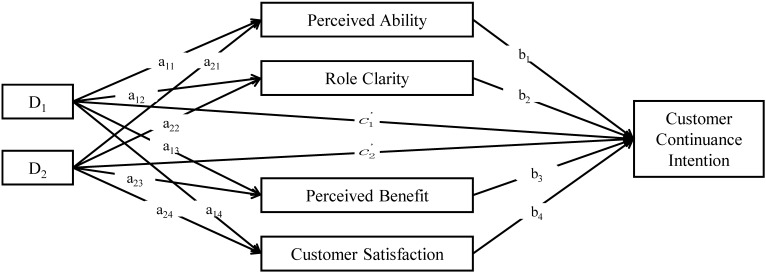
Indirect effects on customer continuance intention.

Prior to the mediation analysis, this study employed an indicator coding strategy to dummy code 3 groups. Two dummy variables were constructed (D*_i_*, *i* = 1, 2), as shown in Figure 2. When a case was in group *i*, the D*_i_* was set to 1; when a case was not, the D*_i_* was set to 0. The low CPSC group was not explicitly coded. (D_1_ and D_2_ were set to 0). In the following analyses, the low CPSC group functions as the reference group, and all estimated parameters that refer to group differences are quantifications in comparison with the reference group (Hayes and Preacher, 2014).

Following the recommendation of Hayes and Preacher (2014), the total effects in this study were quantified with two parameter estimates (c_1_, c_2_) resulting from the estimation of customer continuance intention from the two dummy variable (D_1_, D_2_) coding groups relative to the reference group (i.e., the low CPSC group). The direct effects were quantified with two parameter estimates (c’_1_, c’_2_) from the estimation of customer continuance intention from the two dummy variable coding groups relative to the low CPSC group when mediators were held constant. The indirect effects were represented by the sum of all specific indirect effects through each mediator. All mediation analysis results are shown in Table 4.

**Table 4:** Results of mediation analysis.

	Mediators																				Outcomes				
Antecedents	Perceived ability					Role clarity					Perceived benefit					Customer satisfaction					Customer continuance intention				
	Path	β	SE	LLCI	ULCI	Path	β	SE	LLCI	ULCI	Path	β	SE	LLCI	ULCI	Path	β	SE	LLCI	ULCI	Path	β	SE	LLCI	ULCI
D_1_	a_11_	0.70^∗∗∗^	0.13	0.45	0.95	a_12_	0.54^∗∗^	0.18	0.19	0.89	a_13_	1.03^∗∗∗^	0.11	0.82	1.25	a_14_	0.44^∗∗∗^	0.11	0.22	0.67	*c*’_1_	0.70^∗∗∗^	0.12	0.46	0.94
D_2_	a_21_	1.66^∗∗∗^	0.13	1.40	1.91	a_22_	1.26^∗∗∗^	0.18	0.90	1.61	a_23_	1.83^∗∗∗^	0.11	1.61	2.05	a_24_	1.04^∗∗∗^	0.12	0.81	1.27	c’_2_	0.95^∗∗∗^	0.17	0.62	1.29
Perceived ability																					b_1_	0.17^∗∗^	0.05	0.07	0.27
Role clarity																					b_2_	-0.02	0.04	-0.10	0.05
Perceived benefit																					b_3_	0.14^∗^	0.06	0.02	0.25
Customer satisfaction																					b_4_	0.24^∗∗∗^	0.06	0.13	0.35
Constant		3.90^∗∗∗^	0.27	3.36	4.44		3.74^∗∗∗^	0.38	2.99	4.49		3.16^∗∗∗^	0.23	2.70	3.62		4.51^∗∗∗^	0.24	4.03	4.99		1.71^∗∗∗^	0.40	0.92	2.50

*^∗^p < 0.05; ^∗∗^p < 0.01; ^∗∗∗^p < 0.001.*

The results of the indirect effects analysis (shown in Table 5) indicated that the total indirect effects of moderate CPSC (relative to low CPSC) on customer continuance intention were significant (IE = 0.35, SE = 0.09, 95% CI: [0.19, 0.54]). In addition, the total indirect effects of high CPSC (relative to low CPSC) on customer continuance intention were significant (IE = 0.75, SE = 0.15, 95% CI: [0.46, 1.04]).

**Table 5:** Indirect effects of CPSC on customer continuance intention.

Mediation paths	IE	Boot SE	95% CI
Total indirect effect of moderate CPSC (D_1_)	0.35^†^	0.09	[0.19, 0.54]
Total indirect effect of high CPSC (D_2_)	0.75^†^	0.15	[0.46, 1.04]
Moderate CPSC → *Perceived ability* → Intention	0.12^†^	0.04	[0.05, 0.22]
Moderate CPSC → *Role clarity* → Intention	-0.01	0.03	[-0.09, 0.03]
Moderate CPSC → *Perceived benefit* → Intention	0.14^†^	0.07	[0.01, 0.29]
Moderate CPSC → *Customer satisfaction* → Intention	0.11^†^	0.04	[0.04, 0.22]
High CPSC → *Perceived ability* → Intention	0.28^†^	0.09	[0.12, 0.48]
High CPSC → *Role clarity* → Intention	-0.03	0.06	[-0.16, 0.07]
High CPSC → *Perceived benefit* → Intention	0.25^†^	0.13	[0.02, 0.51]
High CPSC → *Customer satisfaction* → Intention	0.25^†^	0.08	[0.11, 0.42]

^†^*Statistically significant indirect effect.*

Considering specific indirect effects via each of the three mediators, moderate CPSC (relative to low CPSC) had significantly indirect effects on customer continuance intention through perceived ability (IE = 0.12, SE = 0.04, 95% CI: [0.05, 0.22]), perceived benefit (IE = 0.14, SE = 0.07, 95% CI: [0.01, 0.29]), and customer satisfaction (IE = 0.11, SE = 0.04, 95% CI: [0.04, 0.22]). Moreover, high CPSC (relative to low CPSC) had significantly indirect effects on customer continuance intention through perceived ability (IE = 0.28, SE = 0.09, 95% CI: [0.12, 0.48]), perceived benefit (IE = 0.25, SE = 0.13, 95% CI: [0.02, 0.51]), and customer satisfaction (IE = 0.25, SE = 0.08, 95% CI: [0.11, 0.42]). Thus, H2, H4, and H5 were supported. However, the indirect effects of both moderate and high CPSC on customer continuance intention through role clarity were non-significant (CIs straddled zero). Thus, H3 of this research was not supported.

## Discussion

Drawing on insights from the service climate literature and SST research, the present study develops a conceptual framework to investigate the role of CPSC, customer readiness factors, and customer satisfaction in driving customers’ continuance intentions toward in-lobby SSTs in a bank setting. The findings of this empirical study indicate that CPSC affects customer continuance intention directly and indirectly through perceived ability, perceived benefit, and customer satisfaction. Compared with customers who perceive low-level service climate, customers who perceive a high-level service climate have more confidence (i.e., perceived ability) and stronger motivation (i.e., perceived benefit) to continue using in-lobby SSTs. In addition, high-level CPSC leads to greater customer satisfaction, which in turn increases customers’ continuance intentions toward in-lobby SSTs.

All hypotheses of this study are supported except for H3, which deserves comment. The mediation analysis results indicate that role clarity does not play an intervening role in the relationship between CPSC and customer continuance intention. One possible explanation is that the use of SSTs in this study’s context is less frequent and more complicated than other SSTs (e.g., self-checkout kiosks in supermarket) such that customers’ role clarity may be hindered by these traits. Another possible explanation is that perceived ability may have intervened the relationship between role clarity and customer continuance intention, as in the findings of Dong et al. (2008).

### Theoretical Contributions

The current findings yield important theoretical contributions. First, unlike most previous SST studies that focused on customers’ trial intention or initial adoption, this study responds to the call for more research on SST continuance (Meuter et al., 2005) and contributes to a better understanding of customers’ continuance intentions toward SSTs. Second, to the best of our knowledge, this is the first study to link service providers’ managerial practices from the customers’ perspective to customers’ continuance intentions in the SST literature. The extant SST research provides very little knowledge about firm-related variables that drive customers’ continued use of in-lobby SSTs. As an alternative option for interpersonal service, the use of in-lobby SSTs provides opportunities for customers to perceive the service climate, which influences their SST continuance intention. Third, the empirical findings lend support to suggestions proposed by Bitner et al. (2002) for implementing successful SSTs. This empirical study indicates that customers’ perceptions of the service climate are determinants of their continuance intention toward in-lobby SSTs, which is consistent with the proposition that customer-oriented practices (e.g., marketing communication, customer education) are important for both trial and repeated use of SSTs (Bitner et al., 2002).

### Managerial Implications

The current study provides several managerial implications for service firms employing in-lobby SSTs. First, service firms should create a strong service climate to increase customers’ continuance intentions toward in-lobby SSTs. Bowen (1986) theoretically argued that service providers should foster a *climate for service* to improve customers’ role clarity, ability and motivation to participate in service delivery. Furthermore, Bitner et al. (2002) suggested that service firms should actively promote the use of SSTs and maintain a customer focus to implement successful SSTs. The findings of this study are consistent with previous works. Second, service providers should understand that in-lobby service practices, as reflections of service climate, are important to customers’ perceived ability, perceived benefit, and continuance intention toward in-lobby SSTs. Moreover, service firms must be aware of that their role in SSTs encounters is that of a value facilitator rather than a “*bystander.*” When service firms employ in-lobby SSTs, service practices such as marketing communications (e.g., recommendation, promotion), personal assistance (e.g., greeters in bank branches, assistants at airports), clear instructions or demonstrations (e.g., user manual, user guide), and user-friendly interfaces (e.g., ease of use, usefulness) not only influence customers’ initial adoption experience but also determine their continuance intention (Eriksson and Nilsson, 2007; Zhao et al., 2008; Wang et al., 2013). Third, service managers need to highlight the service practices that guarantee customer satisfaction with initial adoption. The mediation analysis results indicate that customer satisfaction is an important mediator in the relationship between CPSC and continuance intention, which is consistent with prior research (Bitner et al., 2002; Chen et al., 2009; Wang, 2012).

## Limitations and Future Research

Although the present study contributes to the understanding of how CPSC affects customer continuance intention toward in-lobby SSTs, it has limitations like any research. First, although the scenario-based experimental approach has been widely used in the service marketing literature, this study encourages future research to employ field experiments. Second, because of the use of scenarios in this study, customer continuance intention was the major dependent variable. However, behavioral intention does not always lead to actual behavior (Sheeran, 2002). Therefore, future research could measure customers’ actual behaviors to improve the findings’ external validity. Third, the present study was conducted in a single service context (i.e., retail bank), which may limit the generalization of the findings. Future research could examine the relationships in multiple contexts. Finally, this study focused on the predicting role of CPSC in SSTs continuance. Future research could explore the potential moderating role of CPSC on the effects of antecedents (e.g., SST characteristics, individual differences) on SST continuance.

## Ethics Statement

We suggest that this study should be exempt from ethics approval. First, this study did not adopt a medical perspective. Second, all customers participated in this study voluntarily. Third, the survey introduction included a data use statement. Participants were notified that the survey data would be collected anonymously, treated confidentially, and used only for scientific publications.

## Author Contributions

ML and SH contributed equally to the work and approved it for publication.

## Conflict of Interest Statement

The authors declare that the research was conducted in the absence of any commercial or financial relationships that could be construed as a potential conflict of interest.

## Appendix

### Basic Scenario

You have a bank card that has not been registered for the online banking service; you want to register it during your lunch break today. You arrive at the nearest bank branch with your bank card. After getting a waiting number, you find that there are 12 people waiting in line ahead of you. Based on your previous experience, you probably will have to wait 1 h for service.

### High CPSC

The lobby manager comes to you and asks you what service you want. He recommends that you use the self-service kiosk in the lobby and gives you a two-page brochure about how to use it. He briefly introduces the operational process and says that he is available to help if you have any problems during the self-service process. It is a user-friendly machine. You complete the online banking registration using it. The entire self-service process takes approximately 15 min.

### Moderate CPSC

Waiting for the service, you find a self-service machine in the lobby. You come to it and find that the online banking registration can be completed by this machine. A two-page brochure about how to use it is placed next to the machine. Following the steps described in the brochure, you complete the online banking registration using the self-service machine. The overall self-service process takes approximately 30 min.

### Low CPSC

Waiting for the service, you find a self-service machine in a corner of the bank lobby. After clicking on the touch screen, you find that the online banking registration can be completed using this machine. You look around and find that there is no user manual or lobby manager who can help. You try to use it for registration on your own. It is an all-in-one machine that has a lot of service functions. After numerous attempts, you finally see “*Registration Complete!*” shown on the screen. The overall service process takes approximately 60 min.

## References

[B1] Ashforth B. E. (1985). Climate formation: issues and extensions. *Acad. Manage. Rev.* 10 837–847. 10.5465/AMR.1985.4279106

[B2] Ashworth W. (2010). *Self-Serve Kiosks: The Payoff Is Huge*. Available at: https://www.investopedia.com/stock-analysis/2010/self-serve-kiosks-the-payoff-is-huge-rst-aapl-mot-gs-mcd-dbt-cstr-bbi-ncr0203.aspx.

[B3] Bagozzi R. P. Yi Y. (1988). On the evaluation of structural equation models. *J. Acad. Market. Sci.* 16 74–94. 10.1007/BF02723327

[B4] Bandura A. (1977). Self-efficacy: toward a unifying theory of behavioral change. *Psychol. Rev.* 84 191–215. 10.1037/0033-295X.84.2.191 847061

[B5] Bhattacherjee A. (2001). Understanding information systems continuance: an expectation-confirmation model. *MIS Quart.* 25 351–370. 10.2307/3250921 28304259

[B6] Bitner M. J. Brown S. W. Meuter M. L. (2000). Technology infusion in service encounters. *J. Acad. Market. Sci.* 28 138–149. 10.1177/0092070300281013

[B7] Bitner M. J. Ostrom A. L. Meuter M. L. (2002). Implementing successful self-service technologies. *Acad. Manage. Execute* 16 96–108. 10.5465/ame.2002.8951333

[B8] Bowen D. E. (1986). Managing customers as human-resources in service organizations. *Hum. Resour. Manage.* 25 371–383. 10.1002/hrm.3930250304

[B9] Bowen D. E. Schneider B. (2014). A service climate synthesis and future research agenda. *J. Serv. Res.-US* 17 5–22. 10.1177/1094670513491633

[B10] Brislin R. W. (1980). “Translation and content analysis of oral and written material,” in *Handbook of Cross-cultural Psychology*, ed. Triandis J. W. B. H. C. (Boston, MA: Allyn and Bacon), 389–444.

[B11] Carr J. Z. Schmidt A. M. Ford J. K. DeShon R. P. (2003). Climate perceptions matter: a meta-analytic path analysis relating molar climate, cognitive and affective states, and individual level work outcomes. *J. Appl. Psychol.* 88 605–619. 10.1037/0021-9010.88.4.605 12940402

[B12] Chen S. C. Chen H. H. Chen M. F. (2009). Determinants of satisfaction and continuance intention towards self-service technologies. *Ind. Manage. Data Syst.* 109 1248–1263. 10.1108/02635570911002306

[B13] Coelho F. J. Augusto M. G. Coelho A. F. Sa P. M. (2010). Climate perceptions and the customer orientation of frontline service employees. *Serv. Ind. J.* 30 1343–1357. 10.1080/02642060802613525

[B14] Curran J. M. Meuter M. L. Surprenant C. F. (2003). Intentions to use self-service technologies: a confluence of multiple attitudes. *J. Serv. Res.-U.S.* 5 209–224. 10.1177/1094670502238916

[B15] Dabholkar P. A. Bagozzi R. P. (2002). An attitudinal model of technology-based self-service: moderating effects of consumer traits and situational factors. *J. Acad. Market. Sci.* 30 184–201. 10.1177/0092070302303001

[B16] Dean A. M. (2004). Links between organisational and customer variables in service delivery: evidence, contradictions and challenges. *Int. J. Serv. Ind. Manage.* 15 332–350. 10.1108/09564230410552031

[B17] Ding X. Verma R. Iqbal Z. (2007). Self-service technology and online financial service choice. *Int. J. Serv. Ind. Manage.* 18 246–268. 10.1108/09564230710751479

[B18] Dong B. B. Evans K. R. Zou S. (2008). The effects of customer participation in co-created service recovery. *J. Acad. Market. Sci.* 36 123–137. 10.1007/s11747-007-0059-8

[B19] Dong B. B. Sivakumar K. Evans K. R. Zou S. M. (2015). Effect of customer participation on service outcomes: the moderating role of participation readiness. *J. Serv. Res.-U.S.* 18 160–176. 10.1177/1094670514551727

[B20] Eriksson K. Nilsson D. (2007). Determinants of the continued use of self-service technology: the case of Internet banking. *Technovation* 27 159–167. 10.1016/j.technovation.2006.11.001

[B21] Evans K. R. Landry T. D. Li P. C. Zou S. (2007). How sales controls affect job-related outcomes: the role of organizational sales-related psychological climate perceptions. *J. Acad. Market. Sci.* 35 445–459. 10.1007/s11747-007-0033-5

[B22] Fornell C. Larcker D. F. (1981). Evaluating structural equation models with unobservable variables and measurement error. *J. Market. Res.* 18 39–50. 10.2307/3151312

[B23] Grönroos C. (2008). Service logic revisited: who creates value? And who co-creates? *Eur. Bus. Rev.* 20 298–314. 10.1108/09555340810886585

[B24] Hayes A. (2013). *Introduction to Mediation, Moderation, and Conditional Process Analysis. A Regression-Based Approach.* New York, NY: Guilford.

[B25] Hayes A. F. Preacher K. J. (2014). Statistical mediation analysis with a multicategorical independent variable. *Br. J. Math. Stat. Psychol.* 67 451–470. 10.1111/bmsp.12028 24188158

[B26] Ho S.-H. Ko Y.-Y. (2008). Effects of self-service technology on customer value and customer readiness: the case of Internet banking. *Int. Res.* 18 427–446. 10.1108/10662240810897826

[B27] Hong Y. Liao H. Hu J. Jiang K. (2013). Missing link in the service profit chain: a meta-analytic review of the antecedents, consequences, and moderators of service climate. *J. Appl. Psychol.* 98:237. 10.1037/a0031666 23458337

[B28] ICBC (2017). *All ICBC Outlets to Realize Intelligent Service in* 2017. Available: http://www.icbc.com.cn/icbc/en/newsupdates/icbc%20news/AllICBCOutletstoRealizeIntelligentServicein2017.htm.

[B29] James L. R. Jones A. P. (1974). Organizational climate: a review of theory and research. *Psychol. Bull.* 81:1096 10.1037/h0037511

[B30] Jin H. J. Yoo J. J. Arnold T. J. (2017). Service climate as a moderator of the effects of customer-to-customer interactions on customer support and service quality. *J. Serv. Res.-U.S.* 20.

[B31] Jones A. P. James L. R. (1979). Psychological climate – Dimensions and relationships of individual and aggregated work-environment perceptions. *Organ. Behav. Hum. Perform.* 23 201–250. 10.1016/0030-5073(79)90056-4

[B32] Kim M. Qu H. (2014). Travelers’ behavioral intention toward hotel self-service kiosks usage. *Int. J. Contermp. Hosp. Manage.* 26 225–245. 10.1108/IJCHM-09-2012-0165

[B33] Lee J. Allaway A. (2002). Effects of personal control on adoption of self-service technology innovations. *J. Serv. Mark.* 16 553–572. 10.1108/08876040210443418

[B34] Lengnick-Hall C. A. (1996). Customer contributions to quality: a different view of the customer-oriented firm. *Acad. Manage. Rev.* 21 791–824. 10.5465/AMR.1996.9702100315

[B35] Lewin K. Lippitt R. White R. K. (1939). Patterns of aggressive behavior in experimentally created “Social Climates.” *J. Soc. Psychol.* 10 271–299. 10.1080/00224545.1939.9713366

[B36] Liao H. Chuang A. (2004). A multilevel investigation of factors influencing employee service performance and customer outcomes. *Acad. Manage. J.* 47 41–58.

[B37] Lin C. H. Shih H. Y. Sher P. J. (2007). Integrating technology readiness into technology acceptance: the TRAM model. *Psychol. Market.* 24 641–657. 10.1002/mar.20177

[B38] Lin J.-S. C. Chang H.-C. (2011). The role of technology readiness in self-service technology acceptance. *Manag. Serv. Qual.* 21 424–444. 10.1108/09604521111146289

[B39] Mayer D. M. Ehrhart M. G. Schneider B. (2009). Service attribute boundary conditions of the service climate–customer satisfaction link. *Acad. Manage. J.* 52 1034–1050. 10.5465/amj.2009.44635617

[B40] Meuter M. L. Bitner M. J. Ostrom A. L. Brown S. W. (2005). Choosing among alternative service delivery modes: an investigation of customer trial of self-service technologies. *J. Market.* 69 61–83. 10.1509/jmkg.69.2.61.60759

[B41] Meuter M. L. Ostrom A. L. Bitner M. J. Roundtree R. (2003). The influence of technology anxiety on consumer use and experiences with self-service technologies. *J. Bus. Res.* 56 899–906. 10.1016/S0148-2963(01)00276-4

[B42] National Bureau of Statistics of China (2017). *Statistical Bulletin of the People’s Republic of China on National Economic and Social Development* 2017. Available: http://www.stats.gov.cn/tjsj/zxfb/201802/t20180228_1585631.html.

[B43] Oh H. Jeong M. Baloglu S. (2013). Tourists’ adoption of self-service technologies at resort hotels. *J. Bus. Res.* 66 692–699. 10.1016/j.jbusres.2011.09.005

[B44] Ostroff C. (1993). The effects of climate and personal influences on individual behavior and attitudes in organizations. *Organ. Behav. Hum. Decis. Process.* 56 56–90. 10.1006/obhd.1993.1045

[B45] Parker C. P. Baltes B. B. Young S. A. Huff J. W. Altmann R. A. Lacost H. A. (2003). Relationships between psychological climate perceptions and work outcomes: a meta-analytic review. *J. Organ. Behav.* 24 389–416. 10.1002/job.198

[B46] Payne A. F. Storbacka K. Frow P. (2008). Managing the co-creation of value. *J. Acad. Market. Sci.* 36 83–96. 10.1007/s11747-007-0070-0 30029666

[B47] Reinders M. J. Dabholkar P. A. Frambach R. T. (2008). Consequences of forcing consumers to use technology-based self-service. *J. Serv. Res.-U.S.* 11 107–123. 10.1177/1094670508324297

[B48] Schneider B. (1973). The perception of organizational climate: the customer’s view. *J. Appl. Psychol.* 57 248–256. 10.1037/h0034724 12002951

[B49] Schneider B. (1975). Organizational climates: an essay. *Pers. Psychol.* 28 447–479. 10.1111/j.1744-6570.1975.tb01386.x

[B50] Schneider B. Ashworth S. D. Higgs A. C. Carr L. (1996). Design, validity, and use of strategically focused employee attitude surveys. *Pers. Psychol.* 49 695–705. 10.1111/j.1744-6570.1996.tb01591.x

[B51] Schneider B. Bowen D. E. (1985). Employee and customer perceptions of service in banks – Replication and extension. *J. Appl. Psychol.* 70 423–433. 10.1037//0021-9010.70.3.423

[B52] Schneider B. Gonzalez-Roma V. Ostroff C. West M. A. (2017). Organizational climate and culture: reflections on the history of the constructs. *J. Appl. Psychol.* 102 468–482. 10.1037/apl0000090 28125256

[B53] Schneider B. White S. S. Paul M. C. (1998). Linking service climate and customer perceptions of service quality: test of a causal model. *J. Appl. Psychol.* 83 150–163. 10.1037/0021-9010.83.2.150 9577232

[B54] Schuster L. Proudfoot J. Drennan J. (2015). Understanding consumer loyalty to technology-based self-services with credence qualities. *J. Serv. Mark.* 29 522–532. 10.1108/JSM-01-2015-2021

[B55] Sheeran P. (2002). Intention–behavior relations: a conceptual and empirical review. *Eur. Rev. Soc. Psychol.* 12 1–36. 10.1080/14792772143000003

[B56] Smith A. K. Bolton R. N. Wagner J. (1999). A model of customer satisfaction with service encounters involving failure and recovery. *J. Market. Res.* 36 356–372. 10.2307/3152082

[B57] Vargo S. L. Lusch R. F. (2004). Evolving to a new dominant logic for marketing. *J. Market.* 68 1–17. 10.1509/jmkg.68.1.1.24036 11499080

[B58] Walker R. H. Johnson L. W. (2006). Why consumers use and do not use technology-enabled services. *J. Serv. Mark.* 20 125–135. 10.1108/08876040610657057

[B59] Wang C. Harris J. Patterson P. (2013). The roles of habit, self-efficacy, and satisfaction in driving continued use of self-service technologies: a longitudinal study. *J. Serv. Res.-U.S.* 16 400–414. 10.1177/1094670512473200

[B60] Wang C. Harris J. Patterson P. G. (2012). Customer choice of self-service technology: the roles of situational influences and past experience. *J. Serv. Manage.* 23 54–78. 10.1108/09564231211208970

[B61] Wang C.-H. (2012). Determinants and consequences of consumer satisfaction with self-service technology in a retail setting. *Manage. Serv. Qual.* 22 128–144. 10.1108/09604521211218945

[B62] Weijters B. Rangarajan D. Falk T. Schillewaert N. (2007). Determinants and outcomes of customers’ use of self-service technology in a retail setting. *J. Serv. Res.-U.S.* 10 3–21. 10.1177/1094670507302990

[B63] Yim C. K. Chan K. W. Lam S. S. K. (2012). Do Customers and employees enjoy service participation? synergistic effects of self- and other-efficacy. *J. Market.* 76 121–140. 10.1509/jm.11.0205

[B64] Zhao X. Mattila A. S. Eva Tao L.-S. (2008). The role of post-training self-efficacy in customers’ use of self service technologies. *Int. J. Serv. Ind. Manage.* 19 492–505. 10.1108/09564230810891923

